# Histological *Ex Vivo* Evaluation of the Suitability of a 976 nm Diode Laser in Oral Soft Tissue Biopsies

**DOI:** 10.1155/2021/6658268

**Published:** 2021-04-28

**Authors:** Gaspare Palaia, Federico Renzi, Daniele Pergolini, Alessandro Del Vecchio, Paolo Visca, Gianluca Tenore, Umberto Romeo

**Affiliations:** ^1^“Sapienza” University of Rome, Department of Oral and Maxillofacial Sciences, Via Caserta 6, Rome 00161, Italy; ^2^Department of Cytology and Cellular Diagnostics, Regina Elena Institute, Via Elio Chianesi 53, Rome 00144, Italy

## Abstract

**Introduction:**

Laser-induced thermal effects can preclude a safe histological evaluation of biopsy resection margins. The aim of this study was to evaluate the suitability of a 976 nm diode laser in oral soft tissue biopsies in an *ex vivo* study.

**Materials and Methods:**

A 976 nm diode laser (Solase®, Lazon Medical Laser, China) has been used in the contact mode, using a 400 *μ*m fiber tip, at different parameters from 4 to 6 W in the continuous wave (CW), with a fluence between 3184 and 4777 J/cm^2^, and pulsed wave (PW) mode, with a fluence between 318,4 and 477,7 J/cm^2^, to obtain 30 samples from fresh pig cadaver tongues. All specimens were subdivided into 6 groups (from A to F), and each group consisted of 5 samples. Two sections were obtained from each sample. A histological analysis was performed using an optical microscope at magnifications of 5x and 10x. Statistical analysis was carried out using Kruskal–Wallis and Dunn's tests.

**Results:**

The results showed that histological readability was optimal in all the samples. The thermal damage was negligible in all groups. The average thermal damage was 208.40 ± 133.81 *μ*m in the epithelial tissue and 330.14 ± 147.45 *μ*m in the connective tissue. The statistical analysis showed no differences between the groups (*p* > 0.05).

**Conclusion:**

A 976 nm diode laser demonstrated good surgical effectiveness that provoked little peripheral damage in the cut edges and allowed a safe histological diagnosis. *Clinical Relevance*. In oral pathology, many times, there is fear in using the laser to remove some lesions due to its thermal effect on the tissues close to the lesion. This effect is always present in the use of the laser, but the intent is to minimize this effect to have as little alteration as possible on the surrounding tissues.

## 1. Introduction

Due to their various advantages, lasers have been recently introduced into dental practice, and they have applications in many fields of dentistry [1, 2]. The most used lasers are the diode (600–980 nm), potassium titanyl phosphate (KTP, 532 nm), carbon dioxide (CO_2_, 10,600 nm), neodymium-doped yttrium aluminium garnet (Nd:YAG, 1064 nm), and erbium-doped yttrium aluminium garnet (Er:YAG, 2940 nm) lasers [3].

As regards the diode laser, it is a versatile tool that finds a wide range of applications such as in soft tissue surgery, periodontology [4], endodontics [5], teeth whitening, and photobiostimulation (PBM) [6].

This kind of laser emits a beam of light that selectively interacts with tissue chromophores such as haemoglobin and melanin to transform light energy into thermal energy. At the point of incidence of the laser beam, the temperature exceeds 100°C, which vaporizes the tissue and generates a haemostatic cut [7, 8]. In this way, it is possible to safely create access incisions (operculectomy, exposure of impacted teeth, and access for cystic lesions), gingivectomies, gingivoplasties, frenectomies, and fornix elongations.

As regards nonsurgical periodontal treatment, the diode laser showed good results if combined with conventional scaling and root planing (SRP), reducing the probing pocket depth, increasing the clinical attachment level, and decreasing the red complex bacteria [9].

Even other oral diseases, such as osteonecrosis of the jaws [10], burning mouth syndrome [11, 12], temporomandibular joint disorder [6], and vascular lesions [13] can be managed using lasers.

However, there is still a debate regarding the possibility to use lasers to make biopsies, and this is because of the surrounding area of the cut in which the laser-induced overheating may create histological artifacts on the resection margins that, especially for clinically suspected lesions, could interfere with the histological reading of the sample, making it difficult to make a clear and safe anatomopathological diagnosis and casting doubts about the true effectiveness of laser biopsies [14, 15].

According to the actual literature, it is always possible to make a diagnosis when performing laser biopsies, but to determine the real extension of the sick tissue, a good operator's skill and knowledge in laser surgery are required [16–18].

Our group has previously tested many other wavelengths that showed encouraging results; the aim of this study was to evaluate the histological effects of a 976 nm diode laser (a wavelength recently introduced on the market) and also to determine the exact extension of the laser-produced histological alterations in order to establish whether it is possible or not to make safe biopsies with such a kind of laser and the clinical protocol to follow to minimize the risk of unreadable samples.

## 2. Materials and Methods

This study was performed *ex vivo* in six swine tongues obtained from animals that had died less than 24 hours before. Pig tongue was chosen because of its similar histological and physiological structures as the human one [7]. A diode laser with a wavelength of 976 nm (Solase®, Lazon Medical Laser, China) and a 400 *μ*m fiber tip in the contact mode was used for this purpose. This laser operates with a frequency of up to 50 kHz, a pulse length between 10 *μ*s and 0.9 s, and a maximum power of 16 W.

Thirty specimens were taken from the tongue dorsum by the same expert operator. They were divided into six groups (A, B, C, D, E, and F) that corresponded to different settings that have been used (Table 1). Groups A, B, and C were treated using a continuous wave (CW) modality at 4 W with a fluence of 3184 J/cm^2^, 5 W with a fluence of 3980 J/cm^2^, and 6 W with a fluence of 4777 J/cm^2^, respectively. In contrast, groups D, E, and F were treated with a pulsed wave (PW) mode at 4 W with a fluence of 318,4 J/cm^2^, 5 W with a fluence of 398 J/cm^2^, and 6 W with a fluence of 477,7 J/cm^2^ (*t*_on_ − *t*_off_: 100 ms–100 ms), respectively. All the samples were fixed in a 10% formalin solution.

Afterwards, 2 sections for each specimen, for a total of 60 sections, were stained with haematoxylin and eosin for histological evaluation performed by a pathologist who was blinded to the purpose of the study (single-blinded study) and measured in *μ*m the extension of the laser-induced thermal effects. The histological analysis was performed using an optical microscope (Leica DM2000) at a magnification of 5x and 10x; the width of thermal damage in the peri-incisional epithelial and connective tissue was measured using Leica Suite 3.4 software.

Finally, a statistical analysis was carried out with the Kruskal–Wallis and multiple comparison Dunn's test using GraphPad Prism 8.4.2 software to understand whether there were statistically significant differences between the groups.

## 3. Results

**Figure 1:**
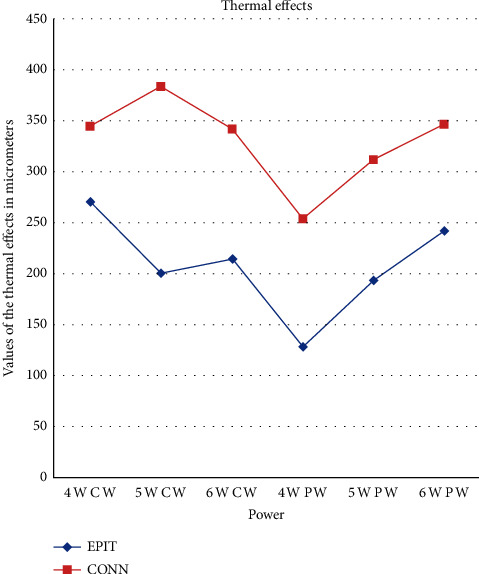
A line chart showing the average values in *μ*m obtained in the epithelium and connective tissue.

**Figure 2:**
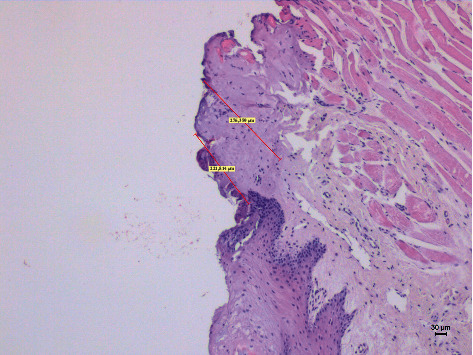
The histological specimen obtained using 4 W in CW with colour EE at 10x magnification (group A).

**Figure 3:**
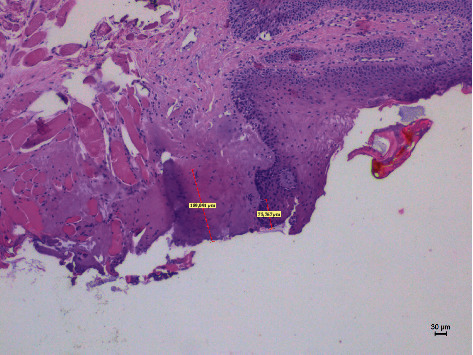
The histological specimen obtained using 4 W in PW with colour EE at 10x magnification (group D).

**Figure 4:**
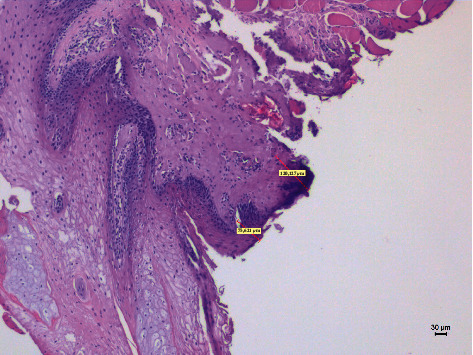
The histological specimen obtained using 5 W in CW with colour EE at 10x magnification (group B).

**Figure 5:**
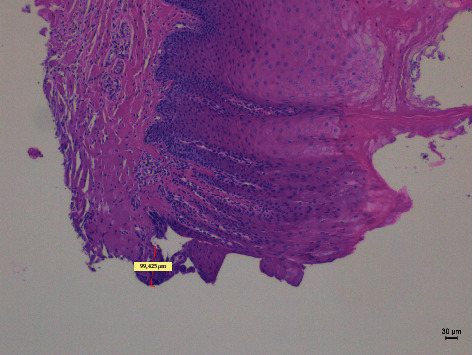
The histological specimen obtained using 5 W in PW with colour EE at 10x magnification (group E).

**Figure 6:**
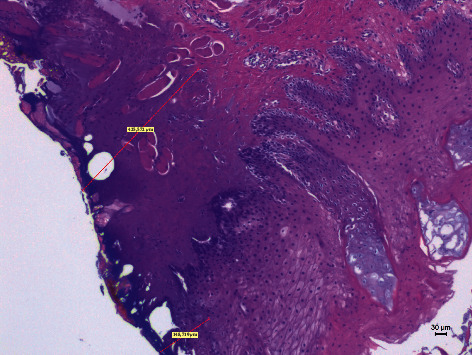
The histological specimen obtained using 6 W in CW with colour EE at 10x magnification (group C).

**Figure 7:**
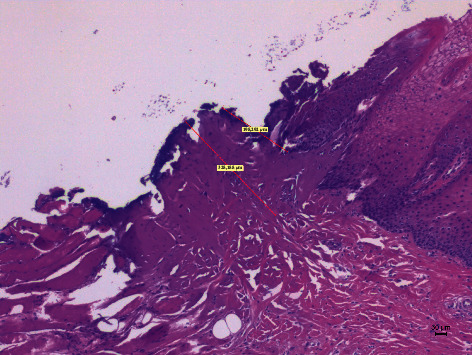
The histological specimen obtained using 6 W in PW with colour EE at 10x magnification (group F).

Different thermal effects emerged in the epithelial and connective tissue layers; the epithelium showed vacuolization, cell coarctation, and carbonization, while collagen homogenization and carbonization were noted in the connective tissue (Figure 1). In the epithelium, the damage extension was always smaller in size, ranging from 46.679 *μ*m to 689.575 *μ*m, compared to the connective tissue, which showed histological alterations from 99.425 *μ*m to 964.24 *μ*m (*p* < 0.01). All samples demonstrated clear and well-readable cut margins with only a small damaged area (Table 2 and Figures 2–7).

The highest damage amounts recorded in this study were 689.575 *μ*m in the epithelium in a sample taken at 6 W in the PW mode and 964.24 *μ*m in the connective tissue in a sample taken at 5 W in the CW mode. No statistically significant differences were found among the groups according to the Kruskal–Wallis and Dunn's tests (*p* > 0.05).

## 4. Discussion

Laser-assisted soft tissue surgery has many advantages over traditional soft tissue surgery for both the operator and the patient. The cut generated by the laser fiber generates hemostasis, because of the affinity of the wavelength for haemoglobin, and contemporary disinfection of the surgical wound due to the laser's photothermal effects. The benefits for the patient include a faster postoperative course, with a faster healing process and less intra- and postoperative pain. In addition, suture is not necessary in many occasions, and this is particularly useful when approaching areas such as the soft palate, where, by healing the tissue for the second intention, the risk of anatomical distortion is minimized [19–22].

However, in the particular case of biopsies, the possibility of using the laser is debated because of the laser's photothermal effects that produce histological artifacts in the marginal portion of the sample that may interfere with the reading of the transition portion between the sick and healthy tissue, and this aspect is critically important in excisional biopsies of clinically malignant lesions [14, 23].

Over time, studies have been carried out to quantify the portion of tissue damaged by the heat produced by the laser both in *in vivo* and *ex vivo* conditions, using different kinds of lasers. Especially, the most recent ones have reported promising results regarding the possibility of carrying out biopsies, with very small peripheral damage that does not affect the diagnosis and that could potentially ensure a correct interpretation of the peripheral margins if used correctly and by a skilled operator.

Studies conducted by Romeo et al. [16–18, 24] using different wavelengths, both in *ex vivo* and *in vivo* conditions, came to the conclusion that all types of lasers tested (diode 980 nm, diode 808 nm, diode 445 nm, Nd:YAG, Er,Cr:YSGG, KTP, and CO_2_) can be used to perform biopsies safely if used properly. Monteiro came to the similar conclusions in a study in which they analyzed the resection margins of fibroepithelial lesions obtained using CO_2_ lasers, diode lasers, Er:YAG, and Nd:YAG *in vivo* compared with the electrosurgical scalpel and cold scalpel, and they assessed that none of the tested lasers showed limitations regarding histological diagnosis [25]. Merigo et al. instead analyzed the temperature increase by using a thermal camera and the histological quality of samples taken through different wavelengths, obtaining positive results in terms of readability and diagnostic reliability [26].

In a study conducted *in vivo* using both KTP and diode laser, it was noted, as was to be expected, that the thermal damage was greater in some lesions such as oral lichen planus characterized by increased cellularity and inflammation compared to lesions such as mucocele that instead suffered minor thermal damage [3].

Angiero et al., in a retrospective study, noted a relationship between the size of the bioptic fragment prelevated with a diode laser and the presence of artifacts. Samples with a size of less than 3 mm led to limitations in diagnosis [27]. Also, Vescovi et al. found that, using a Nd:YAG laser, epithelial and stromal changes were significantly more frequent in specimens with a mean size less than 7 mm (*p* < 0.0001) [28].

The results of our study are in line with those of other reported studies; the size of the thermally damaged area has always been very small (less than a millimetre) both in the epithelium and in the connective tissue, and the peripheral margins were well readable, allowing a good histological evaluation of the samples.

Independent of the setting, the mean thermal damage was 208.40 ± 133.81 *μ*m in the epithelium and 330.14 ± 147.45 *μ*m in the connective tissue. In the PW samples, the average thermal damage was 188.32 ± 137.28 *μ*m in the epithelium and 303.88 ± 129.15 *μ*m in the connective tissue. The thermal damage size was larger in the CW samples, with a mean of 228.47 ± 129.40 *μ*m in the epithelium and 356.39 ± 161.63 *μ*m in the connective tissue. Compared to the CO_2_, KTP, and 445 nm diode lasers in *ex vivo* conditions [7, 17, 18], the 976 nm diode laser generated greater thermal damage but did not compromise the quality of the histological evaluation.

It is interesting to note that, in our study, the damage to the epithelium has always been smaller than that of the connective tissue (*p* < 0.01). This finding is explainable by the selective absorption of the light radiation emitted by the 976 nm diode laser by haemoglobin, which implies not only a greater cutting effectiveness in more vascularized tissues but also greater thermal effects. Therefore, it seems that lasers could work well for obtaining biopsies. However, when using lasers, it is important to be well versed in the physical principles on which they are based to avoid many iatrogenic mistakes.

Based on the data from this study, the PW mode showed better results than the CW one (*p* < 0.05) in the epithelium and therefore is to be preferred allowing the tissue to recover from the thermal shock; moreover, it is important to enlarge the resection margins by about 1–2 mm to avoid that the heat-induced alterations could interfere with the histological evaluation of the transitional mucosa. According to our experience, it is also very important to carefully calibrate the parameters, especially the power (W) and the frequency (Hz). If this is done, it may be possible to safely perform soft tissue biopsies with an optimal histological readability.

## 5. Conclusions

This study showed that a 976 nm diode laser may be safely used in the excision of oral lesions with a suitable width of resection margins. The highest amount of damage recorded was 689.575 *μ*m in the epithelium and 964.24 *μ*m in the connective tissue. No statistically significant differences were found among the groups. We suggest the use of a lower power that allows a good clinical cut in the PW mode to minimize the risk of generating heavy thermal damage in the treated tissue.

Although lasers appear to be an excellent alternative in the treatment of oral benign lesions, they still cannot be considered the first choice in the treatment of suspicious dysplastic or neoplastic lesions. However, their use may not be considered an absolute contraindication for such types of lesions. In these cases, it is particularly important to enlarge the resection margins by about 1 mm compared to the cold blade in order to avoid thermal effects that might disguise the real extent of the pathology and also to use controlled laser settings.

The limitations of this study include that it was conducted *ex vivo*, with all the related limits, and in healthy mucosa. In contrast, pathologic mucosa is characterized by different degrees of inflammation, hyperaemia, and increased cellularity. All these characteristics may affect the laser-tissue interaction and therefore could alter the results obtained.

Further studies, especially *in vivo* investigations, are needed to confirm these results in order to clarify how the anatomopathological alterations of pathological mucosa modify the laser-tissue interaction.

## Figures and Tables

**Table 1:** The laser parameters used in the study.

Samples' number	Power (W)	Group	Mode
5	4	A	
5	5	B	CW
5	6	C	

5	4	D	
5	5	E	PW (*t*_on_ − *t*_off_ 100 ms)
5	6	F	

**Table 2:** The mean peri-incisional effects.

Average thermal effects	
Average epithelial peri-incisional effects of all samples	0.2 ± 0.13 mm
Average connectival peri-incisional effects of all samples	0.3 ± 0.14 mm
Average epithelial peri-incisional effects of PW samples	0.1 ± 0.14 mm
Average connectival peri-incisional effects of PW samples	0.3 ± 0.13 mm
Average epithelial peri-incisional effects of CW samples	0.2 ± 0.13 mm
Average connectival peri-incisional effects of CW samples	0.3 ± 0.16 mm

## Data Availability

The data used to support the findings of this study were supplied by Dr. Daniele Pergolini under license and so cannot be made freely available. Requests for access to these data should be made to Dr. Daniele Pergolini (daniele.pergolini@uniroma1.it).
